# Association of *HLA-G* 3′UTR Polymorphisms with Response to First-Line FOLFIRI Treatment in Metastatic Colorectal Cancer

**DOI:** 10.3390/pharmaceutics14122737

**Published:** 2022-12-07

**Authors:** Lucia Scarabel, Jerry Polesel, Elena De Mattia, Angela Buonadonna, Mario Rosario D’Andrea, Erika Cecchin, Giuseppe Toffoli

**Affiliations:** 1 Experimental and Clinical Pharmacology Unit, Centro di Riferimento Oncologico di Aviano (CRO) IRCCS, Via Franco Gallini n. 2, 33081 Aviano, Italy; 2 Unit of Cancer Epidemiology, Centro di Riferimento Oncologico di Aviano (CRO) IRCCS, Via Franco Gallini n. 2, 33081 Aviano, Italy; 3 Medical Oncology Unit, Centro di Riferimento Oncologico di Aviano (CRO) IRCCS, Via Franco Gallini n. 2, 33081 Aviano, Italy; 4 Ospedale S. Paolo, 00053 Civitavecchia, Italy

**Keywords:** HLA-G, colorectal cancer, immunobiomarker, immune system, personalized medicine, immunocheckpoint

## Abstract

Microenvironmental factors such as non-classical human leukocyte antigen-G (HLA-G) have been associated with cancer invasiveness and metastatic progression. HLA-G expression has been associated with specific single-nucleotide polymorphisms (SNP) in *HLA-G* 3′untranslated region (UTR) in several diseases. The primary aim was to investigate the predictive role of *HLA-G* polymorphisms on treatment efficacy in metastatic colorectal cancer (mCRC) patients homogeneously treated with first-line FOLFIRI (irinotecan, 5-fluorouracil, and leucovorin) and their association with soluble HLA-G (sHLA-G) plasma concentration. *HLA-G* 3′UTR was sequenced in 248 patients. A set of eight polymorphisms and related haplotypes were analyzed for their association with best tumor response, overall survival (OS), and progression-free survival (PFS). sHLA-G was measured by immunoassay in 35 available plasma samples and correlated with *HLA-G* 3′UTR polymorphisms/haplotypes. Our results showed that carriers of rs371194629 (+2960)-Ins allele were at risk for lack of complete response (hazard ratio (HR):0.29, *p_BH_* = 0.0336), while carriers of rs1710 (+3010)-G allele (rs1063320 (+3142)-C allele in linkage-disequilibrium), and rs9380142 (+3187)-G allele had a higher chance of complete response according to additive models (HR:4.58, *p_BH_* = 0.0245; HR:3.18, *p_BH_* = 0.0336, respectively). The combination of rs371194629-Del, rs1710-G, and rs9380142-G alleles forms the UTR1 haplotype. Patients who were carriers of UTR1/UTR-1 diplotype had a greater chance of complete response to therapy (HR:10.59, *p_BH_* = 0.0294). The same three beneficial alleles showed a trend toward higher pre-treatment sHLA-G plasma levels, supporting a functional role for polymorphisms in protein secretion. In conclusion, genetic variants of *HLA-G* are associated with treatment efficacy in mCRC patients treated with first-line FOLFIRI. This finding shed light on the combined effect of this immune system factor and chemotherapy in cancer patients.

## 1. Introduction

Colorectal cancer (CRC) is the third most commonly diagnosed cancer and has risen from third to second place as the leading cause of cancer death worldwide in the last five years [1]. Although the mortality rate of CRC is decreasing in Europe (EUR), it is still the second leading cause of death in 2020 [2]. Approximately 25% of patients with CRC are diagnosed with advanced disease, and another 35% develop metastases during the course of the disease [3]. The typical backbone of first-line chemotherapy for metastatic CRC (mCRC) includes a fluoropyrimidine used in various combinations and schedules with irinotecan (FOLFIRI) or oxaliplatin (FOLFOX or XELOX) [4]. Chemotherapies generally lead to a weakening of a patient’s immune response, resulting in a higher susceptibility to infections and other complications [5]. However, chemotherapies can also induce immunogenic cell death (ICD), a chronic exposure to damage-associated molecular patterns (DAMPs) in the tumor microenvironment (TME) that triggers long-lasting protective immunity against tumors [6,7,8,9]. The interplay between chemotherapy and the immune system could then influence the efficacy of treatment.

To date, several classifications have been published for CRC, including those related to genomics and epigenomics, transcriptomics, TME, and microbiome [10]. In addition, it has been shown that tumor immunocompetence assessment could help stratify patients who benefit from certain therapies [11]. Specifically, Pagès and colleagues demonstrated the prognostic value of the Immunoscore for time to recurrence (TTR), disease-free survival (DFS), and overall survival (OS) in 2681 patients with stage I–III colon cancer by evaluating the total number of tumor-infiltrating T cells and the number of cytotoxic tumor-infiltrating T cells (density of CD3 and CD8 T cell effectors) [12]. Therefore, patients with colon cancer who have a high Immunoscore have the lowest risk of recurrence and the longest survival [12,13]. Any mechanism that directly or indirectly interferes with the immune system could alter the CRC categorization and, thus, the patient’s prognosis and response to treatment [14]. Polymorphisms in immune system factors are one of these mechanisms; therefore, studying the genetic features of the immunological microenvironment could have important clinical implications [15].

Human leukocyte antigen-G (HLA-G) has been suggested as an immunocheckpoint molecule due to its primary tolerogenic role that can counteract the immune response, making it an interesting target for monitoring response to treatment and also for new drug development. Some HLA-G-based strategies have been proposed for cancer immunotherapy, but none of them has reached the market yet [16]. In this light, the study of genetic polymorphisms in *HLA-G* may become an interesting prognostic and predictive biomarker for cancer. To date, few studies have investigated the prognostic value of HLA-G expression and secretion in CRC, and the results have been controversial [17,18,19,20,21]. The GEIA study aims to investigate the impact of HLA-G tumor expression on the efficacy of cancer immunotherapy in patients with solid cancers to evaluate the presence of resistance to the current immunocheckpoint inhibitor strategy (NCT04300088). This study did not address the issue of a possible interaction of HLA-G with chemotherapies received by the patients, which has been highlighted as an interesting aspect to investigate in our recent paper [22]. The impact of *HLA-G* polymorphisms on efficacy and toxicity in stage II-III CRC patients treated with adjuvant fluoropyrimidine-based chemotherapy and also in patients with non-metastatic CRC treated with the FOLFOX4 regimen (folinic acid/5-fluorouracil/oxaliplatin) has been reported previously [23,24]. The aim of this study was to investigate the role of *HLA-G* 3′UTR polymorphisms/haplotypes in response to first-line-FOLFIRI therapy in patients with mCRC and to evaluate the impact on survival and the relation with the soluble HLA-G (sHLA-G) secreted in plasma.

## 2. Materials and Methods

### 2.1. Patients Clinical Data and Study Design

In this retrospective study, we used genomic DNA from blood samples stored at −80 °C in the existing prospective Biobank in the Experimental and Clinical Pharmacology Unit of the Centro di Riferimento Oncologico of Aviano (CRO) IRCCS. Clinical data from 250 eligible patients diagnosed with mCRC were prospectively collected in a pharmacogenetic study by Toffoli and colleagues [25]. The treatment and eligibility criteria have been described previously [25]. Briefly, in the prospective single-arm interinstitutional study, a total of 250 patients with histologically confirmed mCRC, aged 18 to 75 years, a performance status (WHO) of 0 to 2, an absolute neutrophil count and platelet count greater than 2000/µL, and 100,000/µL, respectively, and normal renal and liver conditions, were treated with irinotecan (CPT-11) in combination with 5-fluorouracil (5-FU) and leucovorin (LV) in the first-line setting. More than 90% of patients were treated with the Tournigand-modified FOLFIRI regimen [26], and the others were treated with the FOLFIRI regimen, both based on an intravenous irinotecan dose of 180 mg/m^2^.

In this study, the primary endpoint was the effect of *HLA-G* 3′UTR polymorphisms/haplotypes on the time to the best tumor response to treatment achieved by patients in the course of therapy. Tumor response was assessed by computed tomography scans of measurable lesions at baseline and at least every four cycles. The best tumor response was classified as complete response (CR), partial response (PR), stable disease (SD), or disease progression (PD) according to the WHO criteria [27]. Objective tumor response was defined as (1) complete response (CR vs. SD + PD + PR as reference) and (2) response rate (RR) (CR + PR vs. SD + PD as reference). Treatment response was assessed only in patients who had received at least four cycles of chemotherapy, as previously reported [25]. The secondary endpoint assessed was the effect on OS and progression-free survival (PFS). The OS was defined as the time from the first-drug administration to the last date of follow-up or death, while the PFS was defined as the time from the first-drug administration to the date of first progression/death or last follow-up. In addition, genotype/phenotype association was also assessed as a subgroup analysis. Phenotype was measured as the concentration of sHLA-G in plasma samples, which was obtained as described below. All patients who participated in the study were self-reported to be Caucasian. After approval of the experimental protocol by the Ethical Committee of the coordinator center (CRO—National Cancer Institute, Aviano, Italy), the Institutional Review Board of each participating institution approved the study protocol, which conformed to the ethical guidelines of the 1975 Declaration of Helsinki. All patients gave written informed consent for genetic analysis before participating in the study. All experiments were performed in accordance with the relevant guidelines and regulations [25].

### 2.2. HLA-G Genetic Analyses

Of the 250 patients originally enrolled in the study [25], 248 were available for genetic analysis. Genomic DNA was extracted from peripheral whole blood using the High Pure PCR Template Preparation Kit (Roche Diagnostics GmbH, Mannheim, Germany). We analyzed the most studied germline polymorphisms in the 3′UTR segment of *HLA-G*: rs371194629 (+2960 14-base pair [bp] INDEL), rs1707 (+3003 T > C), rs1710 (+3010 C > G), rs17179101 (+3027 C > A), rs17179108 (+3035 C > T), rs1063320 (+3142 G > C), rs9380142 (+3187 A > G), rs1610696 (+3196 C > G), and rs1233331 (+3227 G > A > T) using the G*01:01:01:01 genomic sequence available on the HLA-G-specific website (http://hla.alleles.org/data/hla-g.html, accessed on 11 July 2018) as reference (release 3.33.0, 11 July 2018). The 3′UTR of the *HLA-G* gene was amplified by polymerase chain reaction (PCR) using the previously published primers HLAG8F: 5′- TGTGAAACAGCTGCCCTGTGT-3′ and HLAG8R: 5′- GTCTTCCATTTATTTTGTCTCT-3′, verified on a 3% agarose gel, and sequenced using the Sanger method as previously described [28,29]. Chromatograms were visualized using Chromas software version 2.01. Genetic data were annotated using the nomenclature published for the coding region of *HLA-G* on the official HLA website (http://hla.alleles.org/data/hla-g.html, accessed on 28 December 2021).

The frequencies of the selected polymorphisms and Hardy–Weinberg equilibrium (HWE) were calculated. A comparison was also performed with the frequencies reported in the public 1000 Genome database (http://www.internationalgenome.org/1000-genomes-browsers/, accessed on 28 December 2021) for the EUR subpopulation according to the human assembly GRCh37 in Ensembl.

*HLA-G* haplotype composition and frequency were determined using the PHASE algorithm version 2.1 [30,31]. The strength of linkage disequilibrium (LD) between pairs of HLA-G markers with MAF > 2% was measured as r^2^ using the freely available software LDPlotter (http://www.pharmgat.org/Tools/pbtoldplotform, accessed on 28 December 2021). The statistic r^2^ < 0.50 indicates low LD, 0.50 ≤ r^2^ < 0.80 moderately high LD, 0.80 ≤ r^2^ < 1 high LD, and r^2^ = 1 perfect LD.

### 2.3. Plasma sHLA-G Analysis

Plasma samples collected from a subset of 35 patients with mCRC prior to the administration of FOLFIRI therapy were available and included in the analysis. A commercially available sHLA-G ELISA kit (Exbio, Biovendor, Vestec, Czechia) was used to measure sHLA-G levels according to the manufacturer’s datasheet. This ELISA assay detects the major HLA-G isoforms: both membrane shedded HLA-G1 and soluble HLA-G5. Absorbance was measured using the Infinite F200 PRO (TECAN, Männedorf, Switzerland) at 450 nm, with the reference wavelength set at 630 nm. All samples were quantified in duplicate, the background was subtracted using the mean absorbance of the dilution buffer control wells, and final sHLA-G concentrations expressed in Units/mL were determined using the four-parameter algorithm with GraphPad version 9. Standard deviations and coefficients of variation (CV) were determined for each sample to evaluate the scatter and precision of the data.

### 2.4. Statistical Analysis

To evaluate the effect of *HLA-G* 3′UTR polymorphisms/haplotypes on the best tumor response to treatment, the cumulative incidence of the best tumor response was calculated through the Kaplan–Meier method for all *HLA-G* 3′UTR polymorphisms with a variant allelic frequency (VAF) ≥ 5% and for all *HLA-G* 3′UTR haplotypes with a frequency of ≥1% in the eligible population [32]. Time at risk was calculated from the date of treatment initiation to the date of best clinical response (CR or CR/PR), date of death, or last follow-up, whichever occurred first. The risk of best tumor response was calculated through the Cox proportional hazards model [32], adjusting for gender, age, site, stage at diagnosis, radical surgery, adjuvant treatment, and number of metastatic sites. To account for competing risk, the Fine and Gray method was applied [33]; differences between Kaplan–Meier estimates were evaluated through Gray’s test [34].

The association between polymorphisms and PFS or OS was evaluated through univariate Kaplan–Meier curves and multivariable Cox proportional hazard models [32]. Differences between Kaplan–Meier estimates were evaluated through the log-rank test. Hazard ratios (HR) and corresponding 95% confidence intervals (CI) were reported for each model. The time at risk was calculated from the date of treatment initiation to progression, death, or last follow-up, whichever occurred first in relation to the outcome under investigation.

A comparison of sHLA-G levels between patients with different 3′UTR *HLA-G* polymorphisms according to genetic models was performed using the Kruskal–Wallis and/or Mann–Whitney test with R software. The significance level was set at *p* < 0.05 (two-sided). To account for multiple comparisons, adjusted *p*-values (i.e., false discovery rate (FDR)) were calculated according to Benjamini and Hochberg method [35]. Statistical analyses were performed using SAS 9.4 and R software (www.r-project.org, accessed on 22 July 2022).

## 3. Results

### 3.1. Patients’ Clinical Data

The main demographic and clinical characteristics of the patients analyzed are shown in Table 1. The median age of these patients was 63.2 years (range: 26.3–75.9), and the median follow-up time was 15.37 months (range: 0.7–63.5 months). The majority of patients with mCRC were classified as stage IV at the time of diagnosis (*n* = 158, 63.7%), received radical surgery (*n* = 198, 79.8%), and 141 (56.8%) patients had more than one metastatic site at the time of enrollment. We observed a preponderance of men (*n* = 161, 64.9%), which is consistent with the global population mCRC incidence [36,37].

### 3.2. HLA-G Genetic Analyses

Among the 9 polymorphisms analyzed, 8 had a VAF of ≥5% and were included in the analysis (rs1233331 had a VAF < 5% and was excluded). All selected polymorphisms had genotype distributions that were consistent with HWE assumptions (*p* > 0.05), although rs371194629 showed a slight deviation (*p* = 0.05) (Supplementary Table S1).

We analyzed the LD between the polymorphisms in the 3′UTR of *HLA-G* and found a complete LD between the rs1710 (+3010 C > G) and rs1063320 (+3142 G > C) polymorphisms (r^2^ = 1) and a moderately high LD between rs371194629 (+2960 14-bp INDEL) and rs1710 (+3010 C > G), rs1063320 (+3142 G > C), rs1610696 (+3196 C > G) (r^2^ = 0.58, 0.58, and 0.60, respectively), and between rs17179101 (+3027 C > A) and rs17179108 (+3035 C > T) (r^2^ = 0.51) (Supplementary Figure S1), consistent with previously reported data [23,24,38]. The results reported for rs1710 (+3010 C > G) must also be considered valid for rs1063320 (+3142 G > C) polymorphism.

The most frequent alleles in our population are the same as those reported in the 1000 Genome Browser for the EUR population, except for the rs1710, rs1063320, and rs9380142 polymorphisms (Supplementary Table S2). The allele frequencies for the rs1710 (+3010 C > G) SNP and for the rs1063320 (+3142 G > C) SNP, in perfect LD with rs1710, differed significantly between our and the EUR population, showing a prevalence of the C allele and the G allele, respectively, in the 248 patients analyzed compared to the EUR population (C allele: *p* = 0.0036, G allele: *p* = 0.0021). In addition, we observed a slight increase in the frequencies of C/C and G/C genotypes in the rs1710 SNP compared to the EUR population (31.5% and 45.97% vs. 20.7% and 50.5%, respectively). Another significant difference in allele frequency was observed for the rs9380142 (+3187 A > G) SNP, with the frequency of the A allele being higher in our mCRC population than in the EUR population (*p* = 0.0222). The rs1610696 (+3196 C > G) SNP showed a trend towards a higher frequency of heterozygous C/G and lower frequency of homozygous C/C genotypes compared to the 1000 Genome Browser EUR population (44.35% and 48.39% vs. 40.2% and 52.3%, respectively). Finally, for the rs371194624 (+2960 14-bp INDEL) polymorphism, a trend toward a higher frequency of the Ins/Ins genotype was observed in our studied population with mCRC compared with the EUR population (19.76% vs. 13.7%). These differences in the frequency of these polymorphisms might reflect a particular molecular pattern in mCRC patients compared with the healthy population.

Considering these 8 variants with VAF ≥ 5%, a total of 8 different 3′UTR *HLA-G* haplotypes were defined by the PHASE algorithm: UTR-1 (DelTGCCCGC), UTR-2 (InsTCCCGAG), UTR-3 (DelTCCCGAC), UTR-4 (DelCGCCCAC), UTR-5 (InsTCCTGAC), UTR-6/18 (DelTGCCCAC), UTR-7 (InsTCATGAC), UTR-13 (DelTCCTGAC) (Table 2). UTR-13 had a frequency of 0.4% (2/496) and was excluded from the analysis. UTR-6 and UTR-18 differ only at one position in the +3227 G > A (rs1233331) polymorphism (G and A, respectively); this SNP was excluded from analysis and consequently from haplotype reconstruction because of its low frequency (<5%). Therefore, UTR-18 was included in the UTR-6 haplotype in our study. The most frequent haplotypes in our mCRC population are UTR-2 (*n* = 146, 29.5%) and UTR-1 (*n* = 140, 28.2%); UTR-3 and UTR-4 have the same frequency of 13.1% (*n* = 65), whereas UTR-7, UTR-5, and UTR-6/18 have the lowest frequencies of 6.5% (*n* = 32), 5.0% (*n* = 25), and 4.2% (*n* = 21), respectively.

### 3.3. Effect of HLA-G 3′UTR Genetic Characteristics on Tumor Response

Multivariable and univariate associations found between each investigated *HLA-G* 3′UTR polymorphism and response variables are summarized in Table 3 and Supplementary Table S3, respectively.

**Figure 1:**
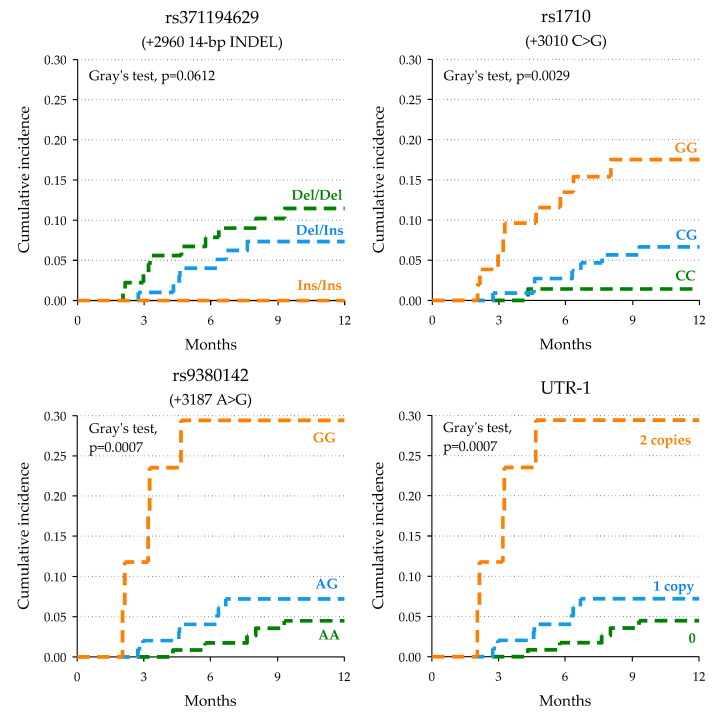
Kaplan–Meier estimate of cumulative incidence of complete response according to selected polymorphisms.

Regarding the impact of *HLA-G* 3′UTR polymorphisms on CR (Table 3), the rs371194629 (+2960)-Ins allele was associated with a lack of clinical response (HR of CR = 0.29, 95%CI: 0.10–0.82, *p_BH_* = 0.0336), with no *Ins/Ins* patients achieving CR versus a 12-month cumulative incidence of 11.5% in *Del/Del* patients (Figure 1). The presence of rs1710 (+3010)-G (HR = 4.58, 95%CI: 1.65–12.72, *p_BH_* = 0.0245) and rs9380142 (+3187)-G (HR = 3.18, 95%CI: 1.25–8.08, *p_BH_* = 0.0336) alleles resulted as a predictive factor for an increased chance of achieving complete response according to additive models (Table 3). Cumulative incidence according to these polymorphisms is shown in Figure 1.

Among the *HLA-G* UTR haplotypes, the UTR-1 haplotype includes all the alleles with a favorable predictive role for complete response (i.e., +2960-Del, +3010-G (+3142-C), and +3187-G). Patients with UTR-1/UTR-1 diplotype had a higher chance of achieving a complete response (HR = 10.59, 95% CI:1.83–61.26, *p_BH_* = 0.0294) compared to those without UTR-1 (Table 3).

### 3.4. Plasma sHLA-G Analysis

**Figure 2:**
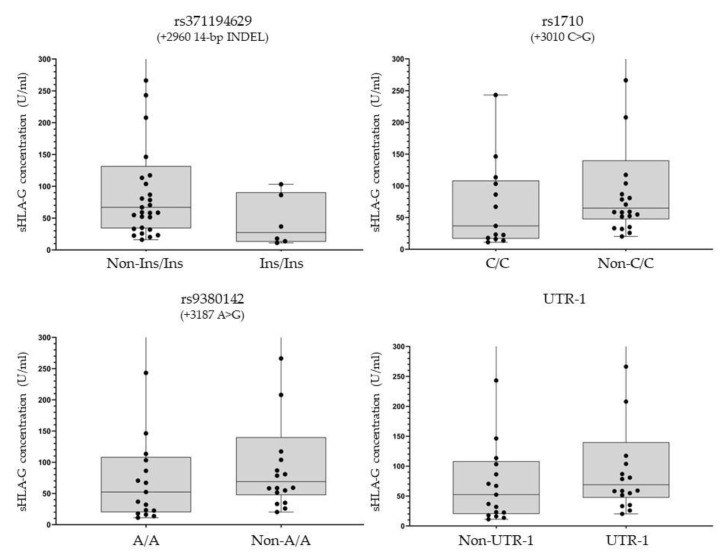
Plasma levels of sHLA-G according to the HLA-G 3′UTR genotypes related to the main HLA-G 3′UTR polymorphisms. The line represents the median value of sHLA-G levels, the grey box the 1st and 3rd quartile values in all the 35 plasma samples analyzed. The group name for rs371194629 refers to patients with Ins/Ins genotype (Ins/Ins) or Ins/Del and Del/Del genotypes (Non-Ins/Ins); for rs1710 to patients with C/C genotype (C/C) or C/G and G/G genotypes (Non-C/C); for rs9380142 to patients with A/A genotype (A/A) or A/G and G/G genotypes (Non-A/A); for UTR-1 haplotype to patients with almost one UTR-1 haplotype (UTR-1) or absence of UTR-1 haplotype (Non-UTR-1).

The median pre-treatment sHLA-G concentration from a subset of 35 available plasma samples was 59.17 U/mL (first-to-third quartile range: 32.60–108.51 U/mL). Focusing on the three *HLA-G* 3′UTR polymorphisms that were significantly associated with tumor response in our study, a trend toward higher pre-treatment sHLA-G levels was observed in the presence of the rs371194629 (+2960)-Del allele (Del/Del+Del/Ins vs. Ins/Ins: 67.1% vs. 27.4%, *p* = 0.0727), rs1710 (+3010)-G allele (64.9% CG+GG vs. 36.9% CC, *p* = 0.1245), and rs9380142 (+3187)-G allele (68.8% AG+GG vs. 52.4% AA, *p* = 0.1760). The same trend was observed for the presence of at least one UTR-1 haplotype (65.3% vs. 52.4%, *p* = 0.1760) (Figure 2).

### 3.5. Effect of HLA-G 3′UTR Genetic Characteristics on Survival Outcomes

The median OS was 15.37 months (range: 0.77–63.50), and overall, 128 deaths (52%) occurred in the 248 patients evaluated in this study during the 5-year observation period. Among the clinical demographic parameters considered, solely the occurrence of radical surgery was significantly associated with OS (HR = 0.44, 95%CI: 0.28–0.68, *p* = 0.0003). After accounting for multiple testing, no significant associations were found in the multivariate analysis between all *HLA-G* 3′UTR polymorphisms/haplotypes and OS.

The median PFS was 7.43 months (range: 0.73–41.60), and 204 out of the 248 eligible patients (89%) documented progressions up to the last date of follow-up. No significant data were found for all *HLA-G* 3′UTR polymorphisms/haplotypes in the overall population. However, focusing on three polymorphisms (rs371194629, rs1710, rs9380142) correlated with clinical response, the rs371194629 (+2960)-Del, rs1710 (+3010)-G, rs9380142 (+3187)-G alleles have a favorable, not statistically significant trend also in the PFS analysis.

### 3.6. Subgroup Survival Analysis: Effect of HLA-G 3′UTR Genetic Characteristics in All Responder Patients

**Figure 3:**
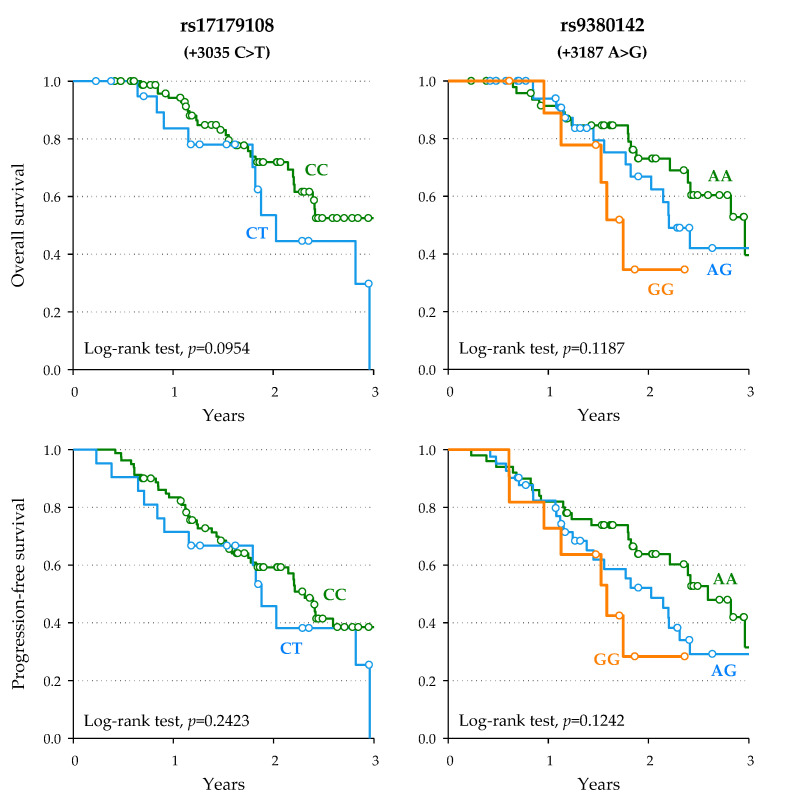
Kaplan–Meier estimate of overall survival and progression-free survival according to polymorphisms +3035 C > T and +3187 A > G. For polymorphism +3035 C > T (rs17179108), only one patient has the TT genotype; therefore, the survival curve is not shown.

A homogeneous subgroup of 102 patients who responded to FOLFIRI chemotherapy was also analyzed for survival outcomes. In this subgroup, which achieved complete or partial response as the best tumor response, rs17179108 (+3035)-T and rs9380142 (+3187)-G alleles were associated with worse OS according to an additive model (HR = 2.37, 95%CI: 1.12–5.01, and HR = 1.95, 95%CI: 1.05–3.61, respectively), though not significant after accounting for multiple testing (*p_BH_* = 0.1173—Table 4, Figure 3). The same detrimental, statistically non-significant trend was observed for PFS (Table 4). Univariate associations are reported in Supplementary Table S4.

**Figure 4:**
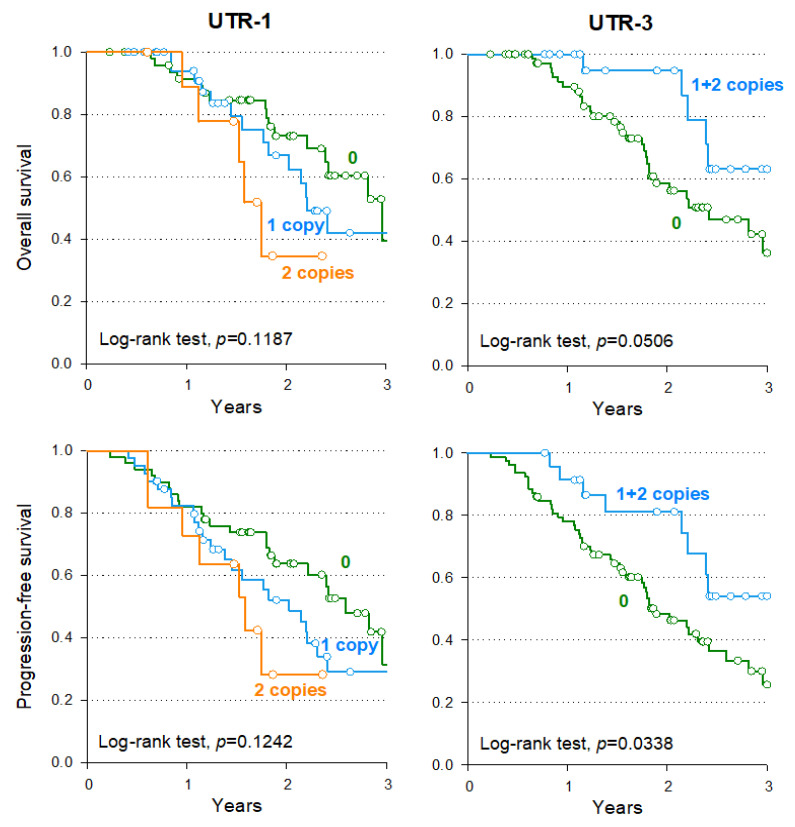
Kaplan–Meier estimates of overall survival and progression-free survival related to the most significant *HLA-G* haplotypes in patients achieving a complete or partial response to treatment.

In this subgroup analysis, rs9380142 (+3187) (G allele) is a tagging SNP for the UTR-1 haplotype, and accordingly, the presence of UTR-1/UTR-1 diplotype was associated with a worse OS (HR = 4.16, 95%CI: 1.17–14.8, *p* = 0.0280) and a non-significantly worse PFS (HR:2.16, 95%CI: 0.81–5.80, *p* = 0.1258) compared to patients who did not carry a UTR-1 allele. Thus, the effect observed for the UTR-1 haplotype on OS appears to be mainly due to the rs9380142 (+3187) (G allele). All these associations lost their statistical significance after accounting for multiple testing. The T allele of rs17179108 (+3035) characterizes the UTR-5 and UTR-7 haplotypes, which have a low frequency in our population (<10%). The presence of at least one copy of the UTR-7 haplotype, including the rs17179101 (+3027)-A (tagging SNP for the UTR-7 haplotype) and the rs17179108 (+3035)-T alleles, and the presence of at least one copy of the UTR-5 haplotype, including the rs17179108 (+3035)-T allele but not the rs17179101 (+3027)-A allele, seems to suggest only the unfavorable effect on OS and PFS. Contrariwise, the presence of at least one copy of the UTR-3 haplotype (all wild-type alleles) showed better OS (HR = 0.25, 95%CI: 0.08–0.75, *p* = 0.0135) and also longer PFS (HR = 0.35, 95%CI: 0.15–0.80, *p* = 0.0129) in multivariate analysis, although the significance was not confirmed after accounting for multiple testing. The Kaplan–Meier curve shows the trend for OS and PFS (Figure 4).

## 4. Discussion and Conclusions

Tumor immunology has focused extensively on local immune responses by studying immunosuppression and modulation of TME, particularly in tissues. However, the interplay with immune responses in the periphery is fundamental to providing a more comprehensive picture of the mechanisms that determine the success of therapies. The interplay of the immune system with conventional chemotherapy and targeted anticancer drugs alters their clinical activity [39]. This suggests that genetic characterization of the immunological microenvironment and immune-related biomarkers of CRC could lead to improved prediction of patients’ clinical response to therapy and prognosis of cancer and also provide novel targets for future drug development.

Since tumor shrinkage is one of the goals to be achieved during treatment, an evaluation of the impact of *HLA-G* 3′UTR polymorphisms on mCRC tumor response was considered. In this retrospective study, we investigated the predictive role of candidate *HLA-G* 3′UTR polymorphisms and haplotypes for the best clinical response to treatment in patients with mCRC treated with irinotecan-based therapy as well as their prognostic role for survival (OS, PFS) in the overall population and in responder patients. Moreover, a protein secretion-related assessment of *HLA-G* polymorphisms was performed by examining their association with soluble HLA-G (sHLA-G) concentration in patients’ plasma.

Our main finding was that three 3′UTR *HLA-G* polymorphisms (rs371194629 (+2960)-Del, rs1710 (+3010)-G, rs9380142 (+3187)-G alleles), and *HLA-G* UTR-1 haplotype, were associated with better response to FOLFIRI therapy in mCRC according to the additive models. Moreover, these clinical/genetic associations were also supported by the peripheral concentration of sHLA-G protein in the plasma of patients before FOLFIRI treatment. In the overall population, no significant effect on survival was found in association with the 3′UTR *HLA-G* polymorphisms. In the homogeneous subgroup of patients who responded to FOLFIRI chemotherapy, the rs9380142 (+3187)-G allele was found to be associated with worse OS, as well as the rs17179108 (+3035)-T allele. Haplotype analysis also indicates that the UTR-1 haplotype is associated with worse OS, whereas the UTR-3 haplotype is associated with better OS and PFS. However, also in this subgroup, these associations were lost after accounting for multiple testing, and therefore, we could conclude that *HLA-G* 3′UTR polymorphisms/haplotypes showed only a trend effect on survival, and other factors could be relevant in this stage of disease.

Specifically, we observed that the rs371194629 (+2960)-*Ins* allele was associated with an increased risk of nonresponse to treatment, whereas the rs1710 (+3010)-G (in complete LD with rs1063320 (+3142)-C) and rs9380142 (+3187)-G alleles were both associated with CR according to additive models. Although no specific regulatory mechanism has been described in the literature, these polymorphic sites could influence mRNA stability. The presence of the rs371194629 (+2960) 14-bp INDEL polymorphism in the *HLA-G* 3′UTR region is widely known to cause mRNA instability and to be involved in the control of post-transcriptional regulation. In particular, the +2960-*Ins* allele confers better resistance to mRNA degradation [40]. The miR-148a and miR-152 downregulate the expression of HLA-G by binding a sequence containing the nucleotide variation at position +3142(rs1063320), regardless of the specific allele present [41]. Other studies reported that the presence of the +3142-*G* allele increases the binding affinity of miRNAs, leading to mRNA degradation and translation suppression [42,43]. As for the +3187 polymorphism, the presence of the A allele leads to decreased HLA-G expression due to the increased number of adenines in the nearby AU-rich motif that mediates mRNA degradation [44].

All significant alleles of the previously reported polymorphisms with a favorable predictive role for complete response (i.e., the +2960-*Del*, +3010-*G* (+3142-*C*), and +3187-*G* alleles) are contained in the *HLA-G* UTR-1 haplotype, which was the only haplotype found to be significantly associated with CR among all *HLA-G* UTR haplotypes. To our knowledge, this is the first time that the UTR-1 haplotype has been associated with complete response in patients with mCRC treated with FOLFIRI.

In general, the *HLA-G* UTR-1 haplotype is one of the haplotypes correlated with higher expression and/or secretion of HLA-G protein. In our mCRC population, the higher sHLA-G concentration found in the plasma of patients with UTR-1-tagging polymorphisms (+2960-*Del*, +3010-*G* (+3142-*C*), +3187-G) suggested a trend consistent with the functional role previously reported in in vitro and in vivo studies [45,46,47].

We also investigated the role of *HLA-G* 3′UTR polymorphisms and haplotypes on OS and PFS in mCRC, but no significant association was found in the entire eligible population. Because the response to treatment is determined by multifactorial factors, including *HLA-G* polymorphisms, stratification in a more homogeneous subgroup of treatment-responsive patients may better highlight prognostic effects due to *HLA-G* polymorphisms. Focusing only on the mCRC patients who respond to FOLFIRI therapy, the presence of the rs9380142 (+3187)-G allele could only suggest a higher risk of death, as is that of the rs17179108 (+3035)-T allele. A non-significant unfavorable trend was observed in patients carrying the rs371194629 (+2960)-Ins/Ins genotype, which was previously associated with higher TNM stage and early risk of relapse in CRC patients [48]. However, all these results were not confirmed after accounting for multiple testing. In addition, the presence of the UTR-1 haplotype may only suggest a worse OS, whereas that of the UTR-3 haplotype (including all wild-type alleles) a favorable survival outcome.

The 5-year survival rate of patients with late-stage CRC in whom cancer has metastasized to distant sites is still about 12–15%, whereas it is about 90% in patients with early-stage CRC [49,50]. In general, the prognostic results we found in metastatic patients are different from those found in non-metastatic patients with stage II-III CRC treated with FOLFOX [23]. The role of the immune system in immunosurveillance usually changes during cancer development, passing from an anti-tumor effector response mediated by T cells secreting Th1 cytokines, NK cell recruitment, and the presence of CTLs, to an immune tolerance response involving immune escape and promotion of immunosuppressive TME [51]. Therefore, the generally poor survival of patients in late-stage CRC could be partly due to an ineffective immune system, which could also affect the response to treatment depending on the phase (elimination, equilibrium, editing, or escape) in the immunosurveillance process. Anticancer drugs, including chemotherapy, could contribute to the alteration of the immunosurveillance process in different ways. We found that treatment was different in the populations analyzed in these two studies. In particular, the adjuvant chemotherapy for early-stage CRC was based on fluoropyrimidine alone or with oxaliplatin [23], whereas the therapeutic regimen for mCRC was FOLFIRI, an irinotecan-based treatment. Therefore, the effects of oxaliplatin and irinotecan might differently modulate the immune system acting on circulating myeloid-derived suppressor cells (MDSCs), with oxaliplatin promoting their depletion and irinotecan their expansion [52]. In addition, it has been previously reported that HLA-G can also modulate the expansion of MDSCs [53].

The results of the present manuscript suggest that changes in the bioavailability of sHLA-G in body fluids determined by the 3′UTR *HLA-G* polymorphism could modulate various mechanisms, including MDSCs expansion, and then affect the interplay between the immune system and FOLFIRI chemotherapy, and influence the patient’s response by a mechanism yet to be elucidated. Moreover, HLA-G also appears to be of interest as a target for its clinical implication and also for the development of novel immunocheckpoint inhibitors due to its immunosuppressive role [54], and some clinical trials are currently underway (NCT04613297, NCT04485013). The exploration of novel immunotherapy strategies has been widespread in recent years, and several immunocheckpoint inhibitors, mainly monoclonal antibodies or small molecules targeting PD-L1, PD-1, or CTLA-4, have emerged as effective anticancer strategies. In particular, pembrolizumab was used for the first time to treat patients with unresectable or mCRC with high microsatellite instability (MSI-H) or mismatch repair defect (dMRR), characterized by upregulated expression of multiple immunocheckpoints.

Some limitations of the study should be mentioned. Because of the retrospective nature of this study, there are patients who were treated without biologic agents that are currently used to improve the efficacy of treatment. However, our entire population was homogeneously treated with the FOLFIRI regimen, which allows us to better investigate the predictive effect of our biomarkers in standard first-line chemotherapy, which is still used today as a typical backbone regimen for CRC. Another limitation is the paucity of plasma samples to correlate genetic data with soluble HLA-G levels. In addition, our data focused on a single immune biomarker, which should also be evaluated considering the contribution of other relevant and emerging prognostic and predictive biomarkers for CRC. This may better capture the complexity of the disease and help explain protein–protein and protein/drug interactions.

In conclusion, HLA-G may be a very interesting molecule to study because of its immunotolerance function and its role in the tumor immune escape mechanism. Evaluation of HLA-G, which is secreted in blood and also expressed in tissue, could support regional (peripheral vs. local) predictive effects and correlation between genetic data and soluble and/or tissue levels of HLA-G could reveal functional hypotheses about the regulatory role of these SNPs. Novel effective anticancer drugs, a deeper knowledge of the molecular features of the disease, the application of personalized, patient-centered strategies, and the development of multidisciplinary teams are necessary to continuously improve the treatment of patients with metastatic and non-metastatic CRC. Additionally, the presence of polymorphisms in immune-related genes cannot be ignored to find the best subgroup of patients who respond to therapy. Indeed, the interaction between the immune system and several types of cancers differently treated can significantly affect the clinical management of patients in terms of predisposition, type, prognosis, and response to treatment of each individual. The overall results for the HLA-G molecule in mCRC treated with the first-line FOLFIRI add value to the management of these patients, which may also have implications from a therapeutic perspective in the field of precision medicine.

## Figures and Tables

**Table 1:** Socio-demographic and clinical characteristics of 248 eligible patients with mCRC treated with first-line FOLFIRI therapy.

Characteristic	*n*	(%)
Gender		
Female	87	(35.1)
Male	161	(64.9)
Age (years)		
<55	62	(25.0)
55–64	85	(34.3)
65–75	101	(40.7)
Cancer site		
Left colon	99	(39.9)
Right colon	78	(31.5)
Rectum	71	(28.6)
Stage at diagnosis		
I–II	25	(10.1)
III	65	(26.2)
IV	158	(63.7)
Radical surgery		
No	50	(20.2)
Yes	198	(79.8)
Adjuvant treatment		
None	161	(64.9)
Chemotherapy	54	(21.8)
Radio-chemotherapy	33	(11.3)
Number of metastatic sites		
1	107	(43.2)
≥2	141	(56.8)
Best clinical response		
Complete response	18	(7.3)
Partial response	84	(33.9)
Stable disease	66	(26.6)
Progression	68	(27.4)
Not evaluated	12	(4.8)
Oncological outcome from treatment initiation	OS	PFS
1 year	77.1%	70.9%
2 years	44.7%	39.5%
3 years	24.5%	19.9%

**Table 2:** *HLA-G* 3′ UTR haplotypes: frequencies distributions in 248 eligible patients with mCRC.

*HLA-G* 3′UTR Haplotypes	+296014-bp	+3003 T>C	+3010 C>G	+3027 C>A	+3035 C>T	+3142 G>C	+3187 A>G	+3196 C>G	Haplotype	Diplotype	
									*n* (%)	Het *n* (%)	Hom *n* (%)
**UTR-2**	**Ins**	T	C	C	C	G	A	**G**	146 (29.5)	109 (44.0)	18 (7.3)
**UTR-1**	Del	T	**G**	C	C	**C**	**G**	C	140 (28.2)	104 (41.9)	18 (7.3)
**UTR-3**	Del	T	C	C	C	G	A	C	65 (13.1)	53 (21.4)	6 (2.4)
**UTR-4**	Del	**C**	**G**	C	C	**C**	A	C	65 (13.1)	53 (21.4)	6 (2.4)
**UTR-7**	**Ins**	T	C	**A**	**T**	G	A	C	32 (6.5)	32 (12.9)	0 (0.0)
**UTR-5**	**Ins**	T	C	C	**T**	G	A	C	25 (5.0)	21 (8.5)	2 (0.8)
**UTR-6/-18**	Del	T	**G**	C	C	**C**	A	C	21 (4.2)	15 (6.0)	2 (0.8)
**UTR-13**	Del	T	C	C	**T**	G	A	C	2 (0.4)	2 (0.8)	0 (0.0)

Abbreviations: Het: heterozygous; Hom: homozygous. The variant allele for each polymorphism was colored in bold/grey.

**Table 3:** Multivariable hazard ratio (HR) and corresponding 95% confidence intervals (CI) ^a^ for clinical response to treatment according to *HLA-G* 3′UTR polymorphisms (additive model) and UTR-1 haplotype.

Alias	SNP rs	CR			CR + PR		
		HR (95%CI)	*p*-Value	*p*-Value_BH_^b^	HR (95%CI)	*p*-Value	*p*-Value_BH_^b^
+2960 Del/Ins	rs371194629	**0.29 (0.10–0.82)**	**0.0192**	**0.0336**	0.87 (0.65–1.15)	0.3171	0.6702
+3003 T > C	rs1707	1.33 (0.54–3.27)	0.6109	0.6109	0.93 (0.64–1.35)	0.7056	0.8538
+3010 C > G	rs1710	**4.58 (1.65–12.72)**	**0.0035**	**0.0245**	1.12 (0.85–1.47)	0.4329	0.6702
+3027 C > A	rs17179101	-	-	-	1.28 (0.68–2.39)	0.4468	0.6702
+3035 C > T	rs17179108	-	-	-	0.94 (0.64–1.39)	0.7589	0.8538
+3187 A > G	rs9380142	**3.18 (1.25–8.08)**	**0.0154**	**0.0336**	1.20 (0.86–1.67)	0.2880	0.6702
+3196 C > G	rs1610696	0.48 (0.15–1.48)	0.2020	0.2357	0.86 (0.62–1.19)	0.3584	0.6702
**Haplotype**	**Patients**	**CR**			**CR + PR**		
		**HR (95%CI)**	***p*-value**	***p*-value_BH_^b^**	**HR (95%CI)**	***p*-value**	***p*-value_BH_^b^**
UTR-1							
0	120	Reference			Reference		
1 copy	99	2.09 (0.70–6.20)	0.1855	0.2357	1.01 (0.67–1.53)	0.9630	0.9630
2 copies	17	**10.59 (1.83–61.26)**	**0.0084**	**0.0294**	1.78 (0.84–3.76)	0.1313	0.6702

Associations with *p*-value < 0.05 are evidenced in bold. ^a^ Estimated from unconditional logistic regression model, adjusting for gender, age, site, stage at diagnosis, radical surgery, adjuvant treatment, and number of metastatic sites. ^b^ Corrected for multiple comparisons according to the Benjamini–Hochberg method.

**Table 4:** Multivariable hazard ratio (HR) and corresponding 95% confidence intervals (CI) ^a^ for death or progression according to *HLA-G* 3′UTR polymorphisms (additive model) and to the most frequent haplotypes in patients with complete or partial response.

SNP	Overall Survival			Progression-Free Survival		
	HR (95% CI)	*p*-Value	*p*-Value_BH_^b^	HR (95% CI)	*p*-Value	*p*-Value_BH_^b^
+2960 Del/Ins	0.94 (0.54–1.62)	0.8147	0.8147	1.02 (0.66-1.47)	0.9299	0.9299
+3003 T>C	0.77 (0.35–1.71)	0.5199	0.6066	0.80 (0.43-1.50)	0.4894	0.5710
+3010 C>G	1.49 (0.91–2.42)	0.1104	0.2151	1.35 (0.91-1.99)	0.1363	0.3103
+3027 C>A	1.74 (0.47–6.43)	0.4066	0.5692	1.53 (0.59-4.00)	0.3820	0.5348
+3035 C>T	**2.37 (1.12–5.01)**	**0.0245**	0.1173	1.68 (0.93-3.04)	0.0876	0.3180
+3187 A>G	**1.95 (1.05–3.61)**	**0.0335**	0.1173	1.46 (0.91-2.33)	0.1130	0.3180
+3196 C>G	0.59 (0.31–1.15)	0.1229	0.2151	0.77 (0.46-1.29)	0.3150	0.5348
Haplotype	**Overall survival**			**Progression-free survival**		
	**HR (95% CI)**	***p*-value**	***p*-value_BH_^b^**	**HR (95% CI)**	***p*-value**	***p*-value_BH_^b^**
UTR-1						
0	Reference			Reference		
1 copy	1.66 (0.63–4.37)	0.3027	0.4793	1.43 (0.70–2.90)	0.3255	0.5557
2 copies	**4.16 (1.17–14.8)**	**0.0280**	0.1330	2.16 (0.81–5.80)	0.1258	0.4780
1 + 2 copies	2.10 (0.88–5.04)	0.0964	0.3097	1.57 (0.81–3.05)	0.1817	0.5557
UTR-2						
0	Reference			Reference		
1 copy	0.63 (0.29–1.38)	0.2436	0.4256	0.78 (0.42–1.45)	0.4364	0.5557
2 copy	0.29 (0.04–2.37)	0.2464	0.4256	0.55 (0.12–2.49)	0.4387	0.5557
1 + 2 copies	0.58 (0.27–1.25)	0.1653	0.3490	0.76 (0.41–1.38)	0.3654	0.5557
UTR-3						
0	Reference			Reference		
1 copy	**0.21 (0.06–0.72)**	**0.0130**	0.0855	**0.34 (0.14–0.83)**	**0.0177**	0.0841
2 copies	0.56 (0.06–4.83)	0.5940	0.7524	0.41 (0.05–3.28)	0.4010	0.5557
1 + 2 copies	**0.25 (0.08-0.75)**	**0.0135**	0.0855	**0.35 (0.15–0.80)**	**0.0129**	0.0817
UTR-4						
0	Reference			Reference		
1 copy	1.12 (0.44–2.85)	0.8066	0.9197	1.09 (0.54–2.23)	0.8061	0.8509
2 copies	-	-		-	-	
1 + 2 copies	0.90 (0.36–2.27)	0.8229	0.9197	0.91 (0.45–1.86)	0.8036	0.8509
UTR-5						
0	Reference			Reference		
1 copy	1.86 (0.50–6.86)	0.3547	0.5184	1.70 (0.60–4.83)	0.3197	0.5557
2 copies	6.19 (0.65–59.4)	0.1141	0.3097	2.37 (0.28–19.94)	0.4257	0.5557
1 + 2 copies	2.33 (0.73–7.44)	0.1535	0.3490	1.80 (0.70–4.65)	0.2240	0.5557
UTR-6						
0	Reference			Reference		
1 copy	**12.95 (2.04–82.2)**	**0.0066**	0.0855	**11.50 (3.60–36.7)**	**<0.0001**	**0.0002**
2 copies	0.91 (0.09–9.09)	0.9324	0.9842	0.63 (0.08-5.37)	0.6754	0.8020
1 + 2 copies	3.16 (0.78–12.8)	0.1075	0.3097	**3.68 (1.41–9.63)**	**0.0078**	0.0741
UTR-7						
0	Reference			Reference		
1 copy	1.74 (0.47–6.43)	0.4066	0.5518	1.53 (0.59–4.00)	0.3820	0.5557

Associations with *p*-value < 0.05 are evidenced in bold. ^a^ Estimated from Cox proportional hazards model, adjusting for gender, age, stage at diagnosis, radical surgery, adjuvant treatment, and number of metastatic sites. ^b^ Corrected for multiple comparisons according to the Benjamini–Hochberg method. Significant associations (*p* < 0.05) are reported in bold.

## Data Availability

The data presented in this study are available on request from the corresponding author. The data are not publicly available due to ethical restrictions.

## Supplementary Materials

The following supporting information can be downloaded at: https://www.mdpi.com/article/10.3390/pharmaceutics14122737/s1, Figure S1: LD patterns at the 3′UTR region of *HLA-G* in 248 patients with mCRC; Table S1: Distribution of polymorphisms and haplotypes of the *HLA-G* 3′UTR region. All selected polymorphisms had genotype distributions consistent with Hardy-Weinberg equilibrium assumptions; Table S2: Frequency distributions of alleles and genotypes identified at *HLA-G* 3′UTR polymorphic sites of 248 patients with mCRC and comparison with those of 503 European (EUR) donors reported in 1000 Genome Browser; Table S3: Univariate hazard ratio (HR) and corresponding 95% confidence intervals (CI) for clinical response to treatment according to *HLA-G* 3′UTR polymorphisms (additive model) and UTR-1 haplotype; Table S4: Univariate hazard ratio (HR) and corresponding 95% confidence intervals (CI) for death or progression according to *HLA-G* 3′UTR polymorphisms (additive model) and to the most frequent haplotypes in patients with complete or partial response.
