# Supplementary material for: Association of HLA-G 3′UTR Polymorphisms with Response to First-Line FOLFIRI Treatment in Metastatic Colorectal Cancer
Source: Pharmaceutics. 2022 Dec 7;14(12):2737. doi: 10.3390/pharmaceutics14122737 (PMC9788252; doi:10.3390/pharmaceutics14122737)
Supplement: Supplementary file 1 [file pharmaceutics-14-02737-s001.zip › pharmaceutics-2040916-supplementary.pdf]

Supplementary materials

Association of *HLA-G* 3′UTR polymorphisms with response to first-line FOLFIRI treatment in metastatic colorectal cancer.

Lucia Scarabel [1], Jerry Polesel [2], Elena De Mattia [1], Angela Buonadonna [3], Mario Rosario D'Andrea [4], Erika Cecchin [1*] and Giuseppe Toffoli [1]

[1] Experimental and Clinical Pharmacology Unit, Centro di Riferimento Oncologico di Aviano (CRO) IRCCS, via Franco Gallini n. 2, 33081 Aviano, Italy
[2] Unit of Cancer Epidemiology, Centro di Riferimento Oncologico di Aviano (CRO) IRCCS, via Franco Gallini n. 2, 33081 Aviano, Italy
[3] Medical Oncology Unit, Centro di Riferimento Oncologico di Aviano (CRO) IRCCS, via Franco Gallini n. 2, 33081 Aviano, Italy
[4] Ospedale S. Paolo, Civitavecchia, Italy
* Correspondence: Author contact info: Centro di Riferimento Oncologico (CRO) IRCCS, via Franco Gallini n. 2, 33081 Aviano, Italy, fax +39-(0)434-659799, email: ececchin@cro.it.

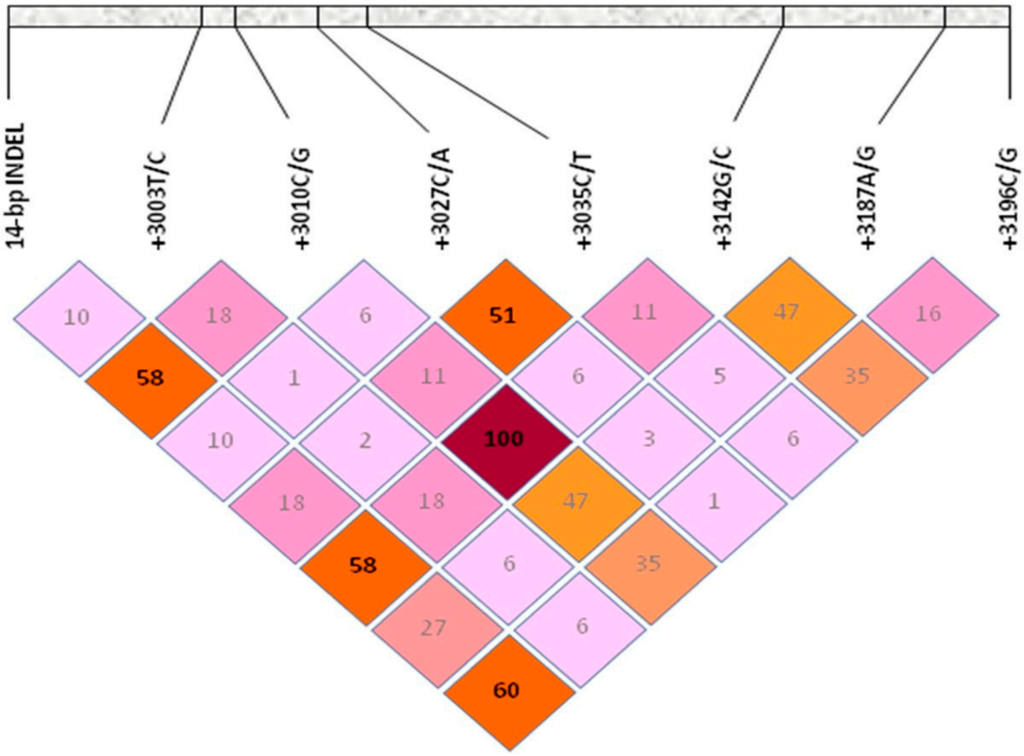

Figure S1. LD patterns at the 3′UTR region of *HLA-G* in 248 patients with mCRC. LD plot generated by LDPlotter shows correlations between all pairs of variants with MAF >2%. The r^2 values (x100) for the marker pairs are listed in the corresponding boxes. High pairwise LD (r^2) between variants is highlighted in bold.

Table S1. Distribution of polymorphisms and haplotypes of the *HLA-G 3'UTR* region. All selected polymorphisms had genotype distributions consistent with Hardy-Weinberg equilibrium assumptions.

	**n**	**(%)**	**MAF**	**HWE**
		496		
+2960 14-bp INDEL (rs371194629)				
Del/Del	94	(37.9)	0.409	p=0.0501
Del/Ins	105	(42.3)		
Ins/Ins	49	(19.8)		
+3003 T>C (rs1707)				
TT	189	(76.2)	0.131	p=0.3317
TC	53	(21.4)		
CC	6	(2.4)		
+3010 C>G (rs1710)[a]				
CC	77	(31.1)	0.456	p=0.3686
CG	116	(46.8)		
GG	55	(22.2)		
+3027 C>A (rs17179101)				
CC	216	(87.1)	0.065	p=0.2774
CA	32	(12.9)		
+3035 C>T (rs17179108)				
CC	194	(78.2)	0.119	p=0.3663
CT	49	(19.8)		
TT	5	(2.0)		
+3187 A>G (rs9380142)				
AA	126	(50.8)	0.282	p=0.5816
AG	104	(41.9)		
GG	18	(7.3)		
+3196 C>G (rs1610696)				
CC	120	(48.4)	0.294	p=0.2863
CG	110	(44.4)		
GG	18	(7.3)		
Haplotype				
UTR1/UTR1	18	(7.3)		
UTR1/UTR2	46	(18.6)		
UTR1/UTR4	20	(8.1)		
UTR2/UTR2	18	(7.3)		
UTR2/UTR3	16	(6.5)		
UTR2/UTR5	10	(4.0)		
UTR2/UTR7	16	(6.5)		
UTR3/UTR1	22	(8.9)		
UTR4/UTR2	17	(6.9)		
Other	65	(26.2)		

[a] +3142 G>C (rs1063320) is in complete linkage disequilibrium with +3010.

Abbreviations: MAF: minor allele frequency; HWE: Hardy-Weinberg equilibrium.

Table S2. Frequency distributions of alleles and genotypes identified at *HLA-G* 3′UTR polymorphic sites of 248 patients with mCRC and comparison with those of 503 European (EUR) donors reported in 1000Genome Browser.

***HLA-G* 3′UTR Polymorphisms**	**Genotypes**	**Our population n (%)**	**EUR n (%)**	**Alleles**	**Our population n (%)**	**EUR n (%)**	**Our population vs. EUR *p*-value***
**+2960 14-bp INDEL**	Del/Del	94 (37.9)	204 (40.6)	Del	293 (59.1)	638 (63.4)	0.1135
(rs371194629)	Ins/Del	105 (42.3)	230 (45.7)	Ins	203 (40.9)	368 (36.6)	
	Ins/Ins	49 (19.8)	69 (13.7)				
**+3003 T>C**	T/T	189 (76.2)	367 (73.0)	T	431 (86.9)	851 (84.6)	0.2454
(rs1707)	T/C	53 (21.4)	117 (23.3)	C	65 (13.1)	155 (15.4)	
	C/C	6 (2.4)	19 (3.8)				
**+3010 C>G**	C/C	77 (31.0)	104 (20.7)	C	268 (54.0)	462 (45.9)	**0.0036**
(rs1710)	G/C	114 (46.0)	254 (50.5)	G	228 (46.0)	544 (54.1)	
	G/G	57 (23.0)	145 (28.8)				
**+3027 C>A**	C/C	216 (87.1)	443 (88.1)	C	464 (93.6)	946 (94.0)	0.7319
(rs17179101)	C/A	32 (12.9)	60 (11.9)	A	32 (6.4)	60 (6.0)	
	A/A	0 (0.0)	0 (0.0)				
**+3035 C>T**	C/C	194 (78.2)	412 (81.9)	C	437 (88.1)	913 (90.8)	0.1219
(rs17179108)	C/T	49 (19.8)	89 (17.7)	T	59 (11.9)	93 (9.2)	
	T/T	5 (2.0)	2 (0.4)				
**+3142 G>C**	G/G	77 (31.0)	104 (20.7)	G	270 (54.4)	462 (45.9)	**0.0021**
(rs1063320)	G/C	116 (46.8)	254 (50.5)	C	226 (45.6)	544 (54.1)	
	C/C	55 (22.2)	145 (28.8)				
**+3187 A>G**	A/A	126 (50.8)	214 (42.5)	A	355 (71.6)	660 (65.6)	**0.0222**
(rs9380142)	A/G	103 (41.5)	232 (46.1)	G	141 (28.4)	346 (34.4)	
	G/G	19 (7.7)	57 (11.3)				
**+3196 C>G**	C/C	120 (48.4)	263 (52.3)	C	350 (70.6)	728 (72.4)	0.4653
(rs1610696)	C/G	110 (44.3)	202 (40.2)	G	146 (29.4)	278 (27.6)	
	G/G	18 (7.3)	38 (7.6)				
**+3227 G>A**	G/G	238 (96.0)	468 (93.0)	G	485 (97.8)	970 (96.4)	0.2066
(rs1233331)	G/A	9 (3.6)	34 (6.8)	A	11 (2.2)	36 (3.6)	
	A/A	1 (0.4)	1 (0.2)				

*calculated with two-sided Fisher's exact test.

Table S3. Univariate hazard ratio (HR) and corresponding 95% confidence intervals (CI)[a] for clinical response to treatment according to *HLA-G* 3′UTR polymorphisms (additive model) and UTR-1 haplotype.

**Alias**	**SNP rs**	**CR**			**CR+PR**		
		**HR (95%CI)**	***p*-value**	***p*-value$_{BH}$[b]**	**HR (95%CI)**	***p*-value**	***p*-value$_{BH}$[b]**
+2960 Del/Ins	rs371194629	**0.41 (0.21-0.78)**	**0.0070**	**0.0138**	0.91 (0.69-1.19)	0.4809	0.7449
+3003 T>C	rs1707	1.35 (0.57-3.18)	0.4972	0.4972	0.94 (0.64-1.37)	0.7310	0.7449
+3010 C>G	rs1710	**3.30 (1.61-6.77)**	**0.0011**	**0.0039**	1.10 (0.84-1.46)	0.4848	0.7449
+3027 C>A	rs17179101	-	-	-	1.22 (0.68-2.19)	0.5099	0.7449
+3035 C>T	rs17179108	-	-	-	0.93 (0.62-1.39)	0.7336	0.7449
+3187 A>G	rs9380142	**2.94 (1.33-6.51)**	**0.0079**	**0.0138**	1.16 (0.85-1.60)	0.3528	0.7449
+3196 C>G	rs1610696	0.60 (0.28-1.28)	0.1896	0.2654	0.92 (0.67-1.25)	0.5733	0.7449

**Haplotype**	**Patients**	**CR**			**CR+PR**		
		**HR (95%CI)**	***p*-value**	***p*-value$_{BH}$[b]**	**HR (95%CI)**	***p*-value**	***p*-value$_{BH}$[b]**
UTR-1							
0	120	Reference			Reference		
1 copy	99	1.71 (0.55-5.31)	0.3522	0.4109	0.93 (0.62-1.41)	0.7449	0.7449
2 copies	17	**8.64 (2.47-30.28)**	**0.0007**	**0.0039**	1.80 (0.95-3.43)	0.0709	0.6381

Associations with *p*-value <0.05 are evidenced in bold.

[a] Estimated from unconditional logistic regression model, adjusting for gender, age, site, stage at diagnosis, radical surgery, adjuvant treatment, and number of metastatic sites. [b] Corrected for multiple comparisons according to Benjamini-Hochberg method.

Table S4. Univariate hazard ratio (HR) and corresponding 95% confidence intervals (CI)[a] for death or progression according to *HLA-G* 3′UTR polymorphisms (additive model) and to the most frequent haplotypes in patients with complete or partial response.

SNP	Overall survival			Progression-free survival		
	**HR (95% CI)**	***p*-value**	***p*-value$_{BH}$[b]**	**HR (95% CI)**	***p*-value**	***p*-value$_{BH}$[b]**
+2960 Del/Ins	1.12 (0.71-1.76)	0.6274	0.6274	1.04 (0.72-1.51)	0.8276	0.8276
+3003 T>C	0.65 (0.31-1.37)	0.2614	0.5329	0.71 (0.39-1.28)	0.2586	0.4526
+3010 C>G	1.16 (0.76-1.77)	0.4850	0.6091	1.23 (0.87-1.74)	0.2361	0.4526
+3027 C>A	1.65 (0.64-4.28)	0.3045	0.5329	1.38 (0.62-3.07)	0.4283	0.5996
+3035 C>T	1.88 (1.00-3.54)	**0.0498**	0.3486	1.46 (0.84-2.53)	0.1806	0.4526
+3187 A>G	1.50 (0.91-2.48)	0.1094	0.3829	1.45 (0.98-2.16)	0.0661	0.4526
+3196 C>G	0.83 (0.47-1.46)	0.5221	0.6091	0.87 (0.55-1.38)	0.5517	0.6437
Haplotype	**Overall survival**			**Progression-free survival**		
	**HR (95% CI)**	***p*-value**	***p*-value$_{BH}$[b]**	**HR (95% CI)**	***p*-value**	***p*-value$_{BH}$[b]**
UTR-1						
0	Reference			Reference		
1 copy	1.22 (0.60-2.50)	0.5821	0.7900	1.39 (0.78-2.48)	0.2610	0.7084
2 copies	2.72 (0.97-7.59)	0.0562	0.3468	2.19 (0.93-5.17)	0.0743	0.2823
1 + 2 copies	1.43 (0.74-2.77)	0.2901	0.6428	1.52 (0.88-2.61)	0.1305	0.4133
UTR-2						
0	Reference			Reference		
1 copy	0.91 (0.47-1.75)	0.7678	0.8143	0.92 (0.53-1.57)	0.7468	0.8347
2 copy	0.47 (0.06-3.57)	0.4680	0.6904	0.63 (0.15-2.67)	0.5337	0.7244
1 + 2 copies	0.86 (0.45-1.65)	0.6582	0.7972	0.89 (0.52-1.51)	0.6561	0.7791
UTR-3						
0	Reference			Reference		
1 copy	0.38 (0.14-1.09)	0.0730	0.3468	0.47 (0.21-1.04)	0.0630	0.2823
2 copies	0.48 (0.07-3.55)	0.4724	0.6904	0.36 (0.05-2.59)	0.3077	0.7244
1 + 2 copies	0.40 (0.16-1.04)	0.0588	0.3468	0.45 (0.21-0.963)	**0.0387**	0.2451
UTR-4						
0	Reference			Reference		
1 copy	0.84 (0.36-1.92)	0.6713	0.7972	0.93 (0.48-1.81)	0.8249	0.8707
2 copies	-		-	-	-	-
1 + 2 copies	0.70 (0.31-1.60)	0.4001	0.6904	0.78 (0.40-1.51)	0.4609	0.7244
UTR-5						
0	Reference			Reference		
1 copy	1.55 (0.55-4.43)	0.4092	0.6904	1.31 (0.52-3.29)	0.5719	0.7244
2 copies	3.30 (0.4-24.67)	0.2445	0.6428	2.03 (0.28-14.85)	0.4847	0.7244
1 + 2 copies	1.74 (0.67-4.94)	0.2544	0.6428	1.39 (0.59-3.26)	0.4504	0.7244
UTR-6						
0	Reference			Reference		
1 copy	9.50 (2.10-43.09)	**0.0035**	0.0665	8.55 (3.22-22.72)	**<0.0001**	**0.0004**
2 copies	0.74 (0.10-5.62)	0.7714	0.8143	0.56 (0.08-4.16)	0.5713	0.7244
1 + 2 copies	2.00 (0.60-6.72)	0.2626	0.6428	2.61 (1.10-6.21)	**0.0294**	0.2451
UTR-7						
0	Reference			Reference		
1 copy	1.65 (0.64-4.28)	0.3045	0.6428	1.38 (0.62-3.07)	0.4283	0.7244

Associations with *p*-value <0.05 are evidenced in bold.

[a] Estimated from Cox proportional hazards model. [b] Corrected for multiple comparisons according to Benjamini-Hochberg method. Significant associations (p<0.05) were reported in bold.
